# Male and female mice display consistent lifelong ability to address potential life-threatening cues using different post-threat coping strategies

**DOI:** 10.1186/s12915-022-01486-x

**Published:** 2022-12-15

**Authors:** Xue Liu, Xiaolong Feng, Hongren Huang, Kang Huang, Yang Xu, Shuwei Ye, Yu-Ting Tseng, Pengfei Wei, Liping Wang, Feng Wang

**Affiliations:** 1 Shenzhen Key Lab of Translational Research for Brain Diseases, Guangdong Provincial Key Laboratory of Brain Connectome and Behavior, CAS Key Laboratory of Brain Connectome and Manipulation, the Brain Cognition and Brain Disease Institute, Shenzhen Institute of Advanced Technology, Chinese Academy of Sciences, Shenzhen-Hong Kong Institute of Brain Science-Shenzhen Fundamental Research Institutions, Shenzhen, 518055 China; 2 University of Chinese Academy of Sciences, Beijing, 100049 China

**Keywords:** Visual stimuli, Innate fear behavior, Sex difference, Lifespan

## Abstract

**Background:**

Sex differences ranging from physiological functions to pathological disorders are developmentally hard-wired in a broad range of animals, from invertebrates to humans. These differences ensure that animals can display appropriate behaviors under a variety of circumstances, such as aggression, hunting, sleep, mating, and parental care, which are often thought to be important in the acquisition of resources, including territory, food, and mates. Although there are reports of an absence of sexual dimorphism in the context of innate fear, the question of whether there is sexual dimorphism of innate defensive behavior is still an open question. Therefore, an in-depth investigation to determine whether there are sex differences in developmentally hard-wired innate defensive behaviors in life-threatening circumstances is warranted.

**Results:**

We found that innate defensive behavioral responses to potentially life-threatening stimuli between males and females were indistinguishable over their lifespan. However, by using 3 dimensional (3D)-motion learning framework analysis, we found that males and females showed different behavioral patterns after escaping to the refuge. Specifically, the defensive “freezing” occurred primarily in males, whereas females were more likely to return directly to exploration. Moreover, there were also no estrous phase differences in innate defensive behavioral responses after looming stimuli.

**Conclusions:**

Our results demonstrate that visually-evoked innate fear behavior is highly conserved throughout the lifespan in both males and females, while specific post-threat coping strategies depend on sex. These findings indicate that innate fear behavior is essential to both sexes and as such, there are no evolutionary-driven sex differences in defensive ability.

**Supplementary Information:**

The online version contains supplementary material available at 10.1186/s12915-022-01486-x.

## Background

A broad range of animal species, including humans, display many sex differences [1–3] from physiological functions to pathological disorders that are developmentally hard-wired and result from sexually differentiated regulation of chromosomes [4, 5], hormones [6–8], neurotransmitters [9], neuronal populations [10–12], neural circuits [13–16], neuroimmunological factors [17], epigenetics and evolutionary processes [18]. There are sex differences in a variety of innate behaviors, such as aggression [19–21], hunting [22], sleep [23], mating [24], and parental care [25], that manifest by sex-dependent regulatory mechanisms across a variety of species. These sexually dimorphic behaviors include a wide range of coordinated and genetically pre-programmed social and sexual displays that ensure the survival of both the individual and the species [8, 18]. Innate behaviors are crucial for the survival and adaptation of the individuals and the species. Innate defensive behaviors are crucial for individual survival in life-threatening circumstances [26]. When faced with potential predators, such genetic programming presumably ensures that animals can instinctively display appropriate defensive behaviors, including when, how, and where to initiate action [27]. Previous studies on neural circuit modulation of innate fear behavior using males and females have not reported sex differences [28–31]. However, it is not well understood whether there is sexual dimorphism in such defensive behavior.

Several innate behaviors, including aggression and sleep, vary with age [19, 32–35]. For instance, mice at postnatal days P-25 and P-35 showed more defensive behavioral inhibition than P-65 adult mice during a fear conditioning session and in response to predatory odor exposure [32]. Likewise, recent studies show that there are age-dependent differences in fear conditioning and upon exposure to aggressive behavior [19–21]. Furthermore, the prevalence of neurodevelopmental [36], neurodegenerative [37], and neuropsychiatric disorders [33] are characterized by sex- and age-dependent differences across lifespan. Whether innate fear behavior is dependent on age in both sexes is still unknown.

In the current study, therefore, we used adult male and female C57BL/6 J mice to study whether visually-triggered defensive behavior is sex-dependent. By employing a 3 dimensional (3D)-motion learning framework developed recently by our group [38], we analyzed the hierarchical dynamics of visually-evoked defensive behaviors. We found that innate defensive responses for life-threatening stimuli are indistinguishable in both sexes throughout the lifespan. Moreover, we reveal a subtle sexually divergent expression of innate fear behavior in terms of architecture and strategy. Our results indicate that, although visually-evoked innate fear behavior is highly conserved throughout life in both sexes, sexually divergent post-threat coping strategies may help males and females to appropriately respond to different external environments. These findings may provide a complementary insight into understanding conserved innate fear behavior.

## Results

### Male and female mice exhibit indistinguishable innate fear behavior in response to potential life-threatening cues

**Fig. 1:**
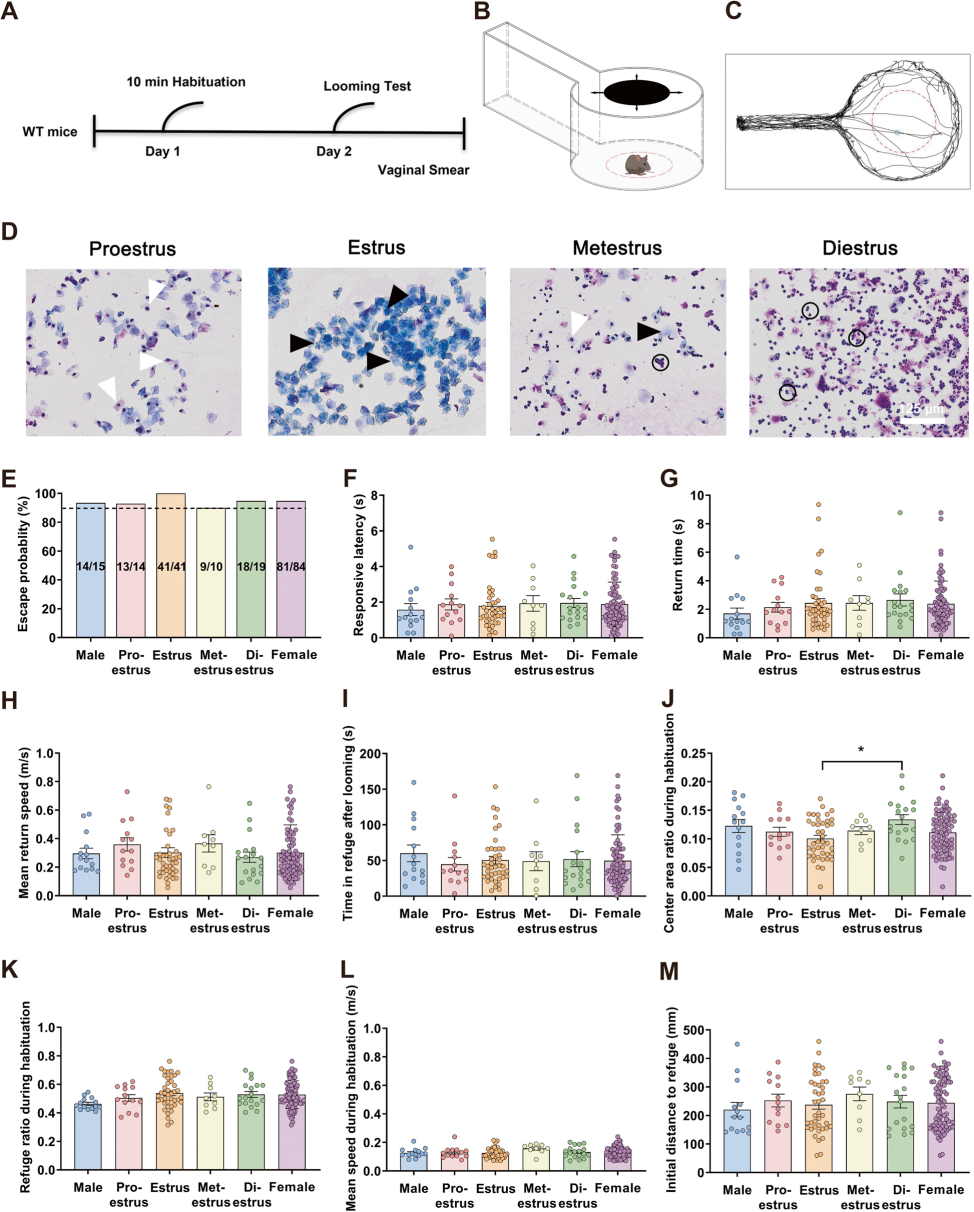
Male and female mice have comparable ability to successfully address approaching sky predators. **A** Experimental flow chart. **B** Schematic showing the experimental setup: a cylindrical open field, a rectangular refuge, and an infrared touchscreen frame below. Visual looming stimuli were presented automatically in the open field. **C** Representative movement trajectory in the looming stimuli setup. The red dotted circle represents the center, within which looming stimuli can be triggered. The small blue circle represents a representative sample of the location where a mouse was exposed to looming stimuli. **D** Vaginal cytology representing each stage of the estrous cycle. Three cell types were identified: leukocytes (circle), cornified epithelial (black arrow), and nucleated epithelial (white arrow). Estrous cycle stages include proestrus, estrus, metestrus, and diestrus from left to right. **E**–**M** Between-group comparisons of **E** escape percentage following looming stimuli, **F** the latency to initiate flight behavior following looming stimuli onset, **G** the latency to return to the refuge following looming stimuli onset, **H** the mean speed of return to the refuge following looming stimuli onset, **I** the time spent in the refuge following looming stimuli onset, **J** the ratio of time spent in the center during the 5-min acclimation period, **K** the ratio of time spent in the refuge during the 5-min acclimation period, **L** the mean speed during the 5-min acclimation period to the arena, and **M** the distance of initial location at stimuli onset to refuge following looming stimuli onset. *p < 0.05. Scale bar, 125 μm. Data are expressed as mean ± SEM

In natural environments, visual signals are an essential sensory input for animals under both non-threatening and threatening conditions for detecting and avoiding salient dangers, such as approaching predators or colliding objects. In this study, a looming stimulus test was used to investigate sex differences in innate fear behavior. The looming test was performed using an automatic behavior detection system to quantify innate defensive behaviors in a circular open-field arena [39, 40] (Fig. 1A, B; Additional file 1: Movie S1). Vaginal smears were analyzed to identify the estrous phase in female mice immediately after the looming test (Fig. 1A, D). Mice were automatically presented with a one-trial looming stimulus when they entered the center of the open field, an unmarked circular area concentric with the circular arena [39]. Looming stimuli triggered flight behavior in ~ 90% of mice (Fig. 1E), and there were no sex or estrous phase differences in responsive latency, return-to-refuge time, mean return speed, or time spent in the refuge after looming stimuli (Fig. 1F–I). We also analyzed spontaneous behavior during a 5-min acclimation period before the looming stimulus and found that there were no differences between sexes in time spent in the central area, time spent in the refuge, and mean speed (Fig. 1J–L). However, we found that female mice in the diestrus phase spent more time in the central area than those in the estrus phase (Fig. 1J) during the 5-min acclimation period. In addition, there were no sex or estrous phase- differences in the initial distance traveled to refuge (Fig. 1M). These results suggest that flight induced by upper-visual-field looming stimuli is highly consistent in both sexes.

To further explore the impact of the estrous cycle on innate fear behavior induced by visual-threat stimuli in females, we conducted bilateral ovariectomies to eliminate the estrous cycle. Vaginal smears were analyzed from day 15 to day 19 to verify that ovaries had been excised successfully before the looming test was performed at day 31 (Additional file 2: Figure S1A). Female mice that underwent ovariectomy (Additional file 2: Figure S1B) no longer displayed periodic changes [41] (Additional file 2: Figure S1C). There was no significant difference in the visual-stimuli-evoked innate fear in castrated females compared to sham females or males, and neither was there a difference during spontaneous behavior during the 5-min acclimation period (Additional file 2: Figure S1D-K). These results indicate that elimination of the estrous cycle in female mice does not affect the ability to successfully address visual life-threatening stimuli.

Animals exhibit innate defense behaviors in response to approaching threat cues by the dynamics of various sensory input modalities, such as visual input from approaching aerial predators [42], predator odors [43], and warning sounds [44, 45]. To investigate sexual dimorphism in defensive behavior across a sensory modality other than vision, we tested auditory-induced defensive behavior. Consistent with previous research [29], we observed moderate defensive behavior in both sexes. Following a 5-min acclimation period, a wideband noise (80 dB sound pressure level, 5-s duration) delivered from a speaker in the arena was triggered automatically (Additional file 3: Figure S2A and Additional file 4: Movie S2), resulting in escape behavior in each animal tested away from the sound to the refuge (Additional file 3: Figure S2B). This included males, estrus-phase females, and non-estrus-phase females (including proestrus-, metestrus-, and diestrus-phase females). In addition, there were no sex or estrous-phase differences in the initial distance to refuge (Additional file 3: Figure S2C). Moreover, there were no sex differences or estrus-phase differences in response latency, return-to-refuge time, mean return speed, or in time spent in the refuge after auditory stimuli (Additional file 3: Figure S2D-G), indicating that the ability to address life-threatening stimuli processed through different sensory modes is of equal importance for both sexes.

### Sex differences in visually-evoked defensive behavior architecture and phenotypes captured using a 3D motion-capture system

**Fig. 2:**
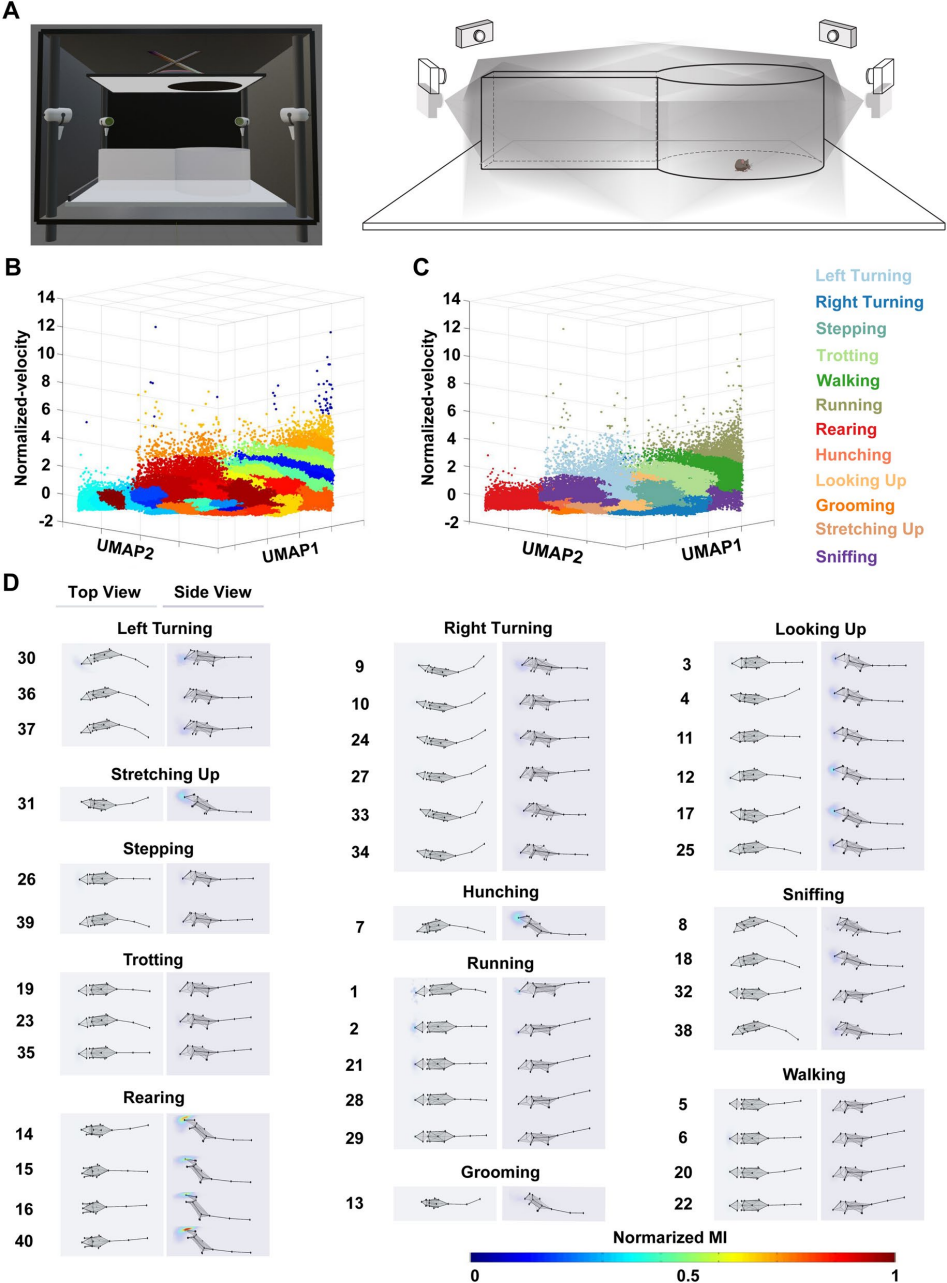
The 3D-motion multi-layered framework adapted for the visually-evoked defensive behavior paradigm. **A** Schematic showing the experimental setup (left) and schematic diagram of the behavioral recording arena with four synchronized cameras (right). **B** Spatiotemporal feature space of behavioral components with unsupervised learning. **C** Spatiotemporal feature space of behavioral components. **D** Average skeleton positions from all frames within each movement phenotype. Skeletons are shown with solid lines and calculated by averaging poses of body parts over time. The heatmaps overlaid on the average noses position correspond to the normalized moving intensity (MI) of each movement phenotype

**Fig. 3:**
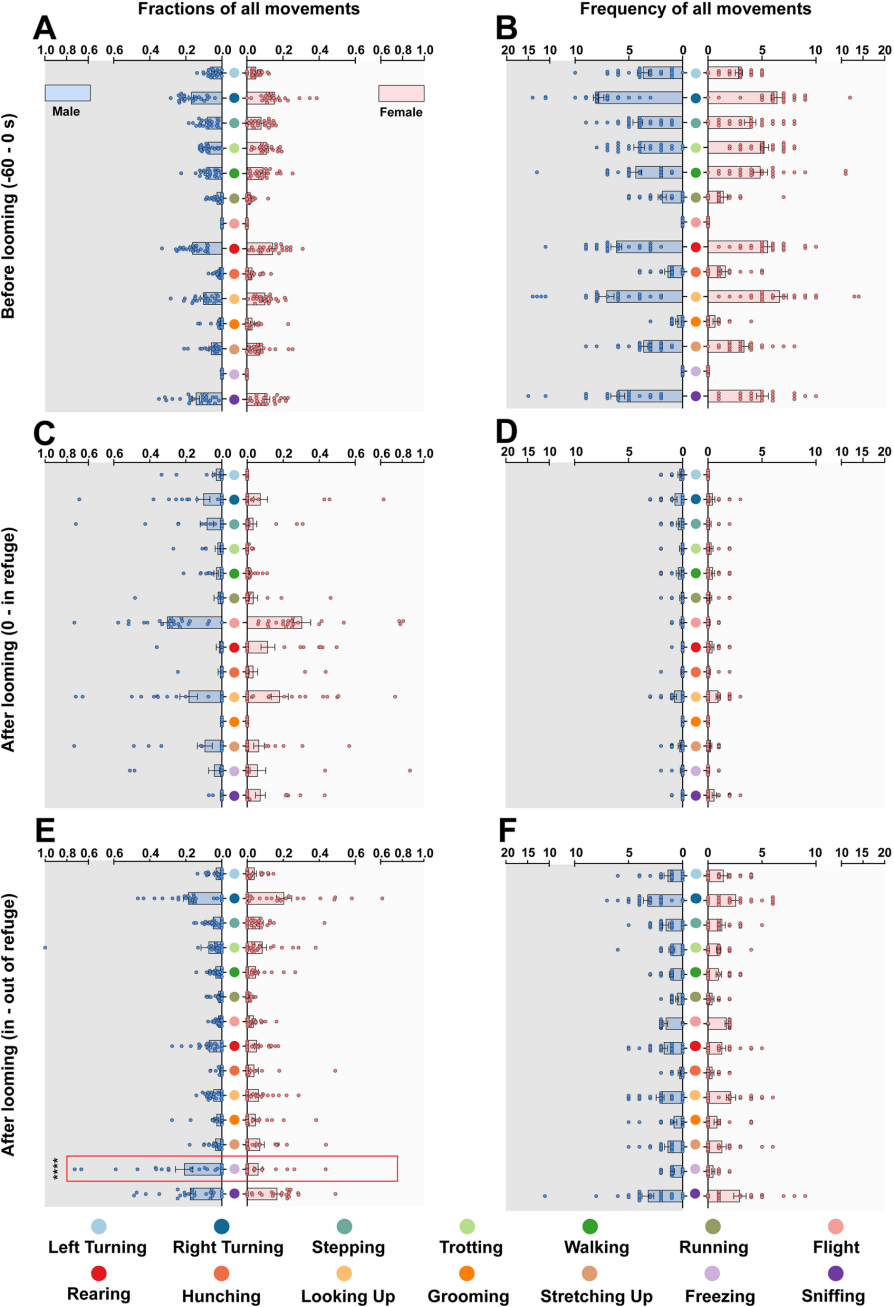
Behavioral category clusters of movements made during the 60 s before looming stimuli to the time at which the mouse left the refuge. Comparison of **A** the fraction and **B** frequency of movement types between male and female mice from − 60 s before looming stimuli to the looming stimuli timepoint (0 s). Between-sex comparisons of **C** the fraction and **D** frequency of movement types from the looming stimuli timepoint (0 s) to the timepoint at which the mouse went into the refuge. Between-sex comparisons of **E** the fraction **F** and frequency of movement types from the timepoint at which the mouse went into the refuge to the timepoint at which the mouse left the refuge. A total of 14 behavioral categories were clustered. The fractions and frequency of each group; blue or red color traces represent the fractions from 45 mice (blue, male, *n* = 23 mice; red, female, *n* = 22 mice). Bottom color-coded labels and dendrogram indicate the movement types. ****p < 0.0001. Data are expressed as mean ± SEM

**Fig. 4:**
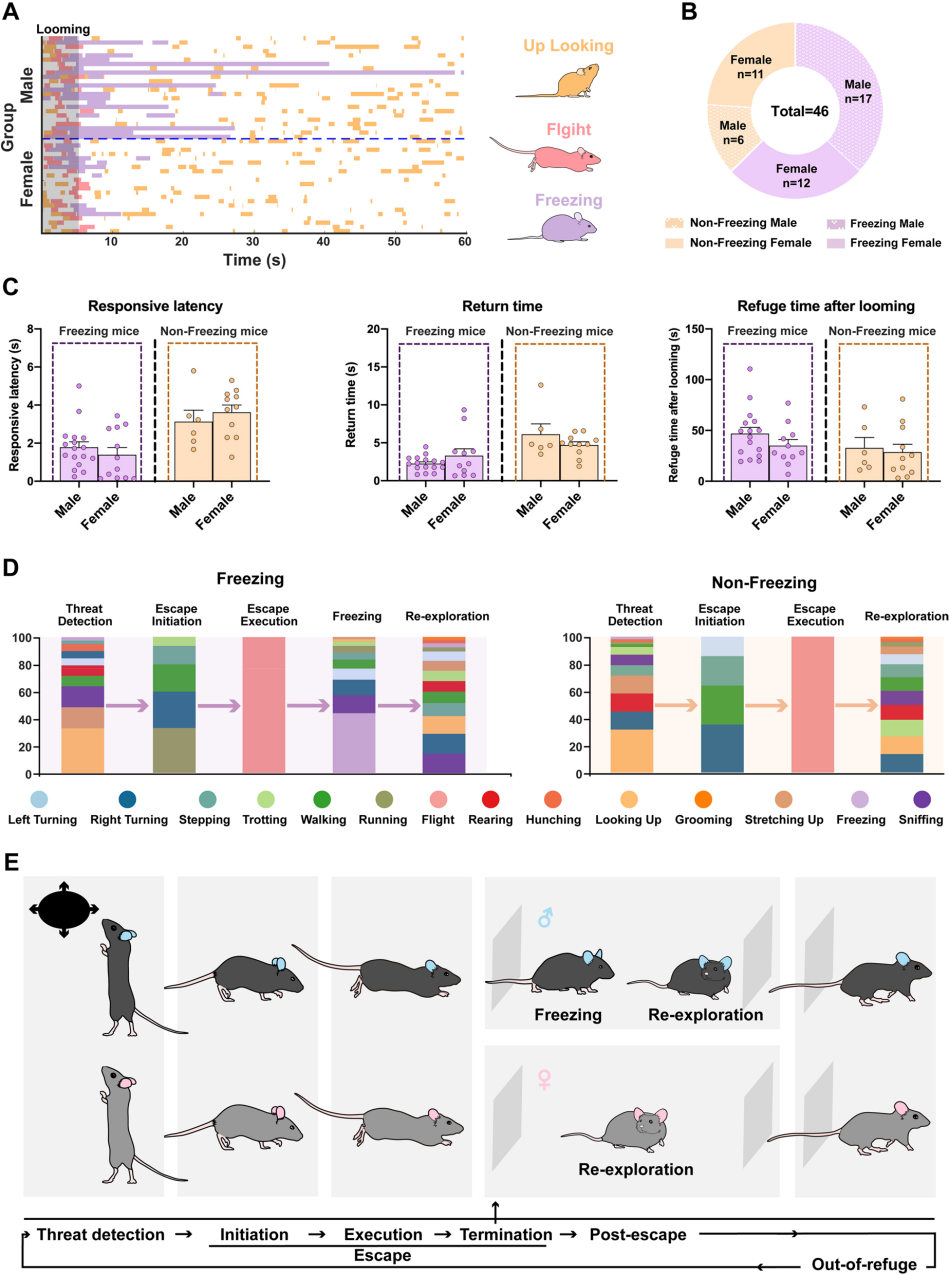
Both sexes are consistently able to successfully address potential life-threatening cues using different post-threat coping strategies. **A** Ethograms of the three defensive movements, looking up, flight and freezing. **B** The number of Freezing and Non-Freezing group mice in both sexes. **C** The two mouse sub-groups displayed similar defensive behavior following looming stimuli. **D** Action sequences and ethograms of mice in the two sub-groups. **E** Schematic diagram showing the conceptual timeline of events during escape behavior. Data are expressed as mean ± SEM. The fractions of each group and light color traces represent the fractions from 46 mice (blue, male, *n* = 23 mice; red, female, *n* = 22 mice)

To quantify whether there were sex differences in more comprehensive movement phenotypes following visually threatening stimuli, we took advantage of the multi-layered framework developed recently by our colleagues [38] and obtained behavioral phenotypes from both sexes (Fig. 2B–D). Behavioral data from 45 12-week-old adult mice (male = 23, female = 22) were collected using a multi-view video capture device (Fig. 2A) from − 60 s before looming stimuli to the timepoint at which the mouse left the refuge after looming stimuli (Additional file 5: Movie S3, Additional file 6: Movie S4). The data were automatically analyzed using Behavior Atlas software developed by our laboratory group (Additional file 7: Figure S3), which yielded 40 behavioral phenotypes with unsupervised clustering (Fig. 2B, D). The 3D feature space was composed of two non-locomotion dimensions and one locomotion dimension and showed good quality unsupervised clustering (Fig. 2B). We obtained 12 behavioral movements (Additional file 8: Table 1) from 45 animals by manually designating each behavioral phenotype, and the 3D feature space showed good quality supervised clustering (Fig. 2C). After reviewing the 40 phenotypes, we designated movements 30, 36, and 37 as left turning, movements 9, 10, 24, 27, 33, and 34 as right turning, movements 3, 4, 11, 12, 17, and 25 as looking up, movement 31 as stretching up, movements 26 and 39 as stepping, movement 7 as hunching, movements 8, 18, 32, and 38 as sniffing, movements 19, 23, and 35 as trotting, movements 1, 2, 21, 28, and 29 as running, movements 5, 6, 20, and 22 as walking, movements 14,15, 16, and 40 as rearing, and movement 13 as grooming (Fig. 2D). Furthermore, we also recognized two classical defensive movements during this period, namely, flight and freezing (Figs. 3 and 4A, Additional file 8: Table 1).

A conceptual timeline of escape events during defensive behavior consists of threat detection, escape initiation, escape execution, and escape termination [27]. Nevertheless, behavior after escape termination has not been well-studied. Our data showed that there were no sex differences in the frequency or in the fractions (the proportion of movements made relative to the entire time period) of any movements, including flight and freezing, from threat detection to escape termination (-60 s- refuge reached) (Fig. 3A, B). Moreover, in this period, there were no significant between-sex differences in mean and maximum speed (Additional file 9: Figure S4A, D-H), or in distance and duration (Additional file 9: Figure S4B-C). However, there was a higher proportion of freezing in male mice than in female mice when in the refuge after escape termination (Fig. 3E), although there was no sex difference in freezing frequency overall. It is remarkable that we did not observe freezing following looming stimuli in all mice (Fig. 4A, B). We classified mice into two groups based on freezing behavior: those that presented freezing-like behavior following looming stimuli were in the Freezing group and those that did not were in the Non-Freezing group. Over 70% of males displayed freezing compared to only approximately 50% of females (Fig. 4B).

Further, we performed dimensionality reduction to visualize the behavioral map to facilitate exploration of the evolution of high-order movement sequences and behavioral-state transitions (Additional file 9: Figure S4I) caused by visually-evoked innate defensive behavior. For this process, we used the Freezing and Non-Freezing sub-groups. There was no sex difference in flight behavior between the two sub-groups (Fig. 4C). However, females had a smaller proportion of freezing fractions than males after entering the refuge (Fig. 3E), indicating that female mice tended towards “active” or “positive” behaviors compared to male mice after termination of the escape state. Therefore, we propose a post-escape period following escape termination as part of the whole defensive behavioral process. In this period, there are two different behavioral features: (1) freezing and then re-exploration and (2) direct re-exploration (Fig. 4D, E). Action sequences in mice of both sexes in the Freezing sub-group (threat detection, escape initiation, escape execution, freezing, and re-exploration) and the Non-Freezing sub-group (threat detection, escape initiation, escape execution, and re-exploration) can be seen in Fig. 4D, E. Among them, the threat detection period mainly included looking up, hunching, sniffing, walking, rearing, and turning. The pre-escape period consisted of walking, stepping, right turning, and running. All mice displayed flight during the escape period. Post-escape movement architecture included freezing, sniffing, turning and others (Fig. 4D). Together, our data using a 3D motion-capture system indicate that both sexes are equally capable of addressing life-threatening cues using different post-threat coping strategies when presented with visually-threatening stimuli.

### Visually-evoked innate fear behavior is constant over the mouse lifespan

**Fig. 5:**
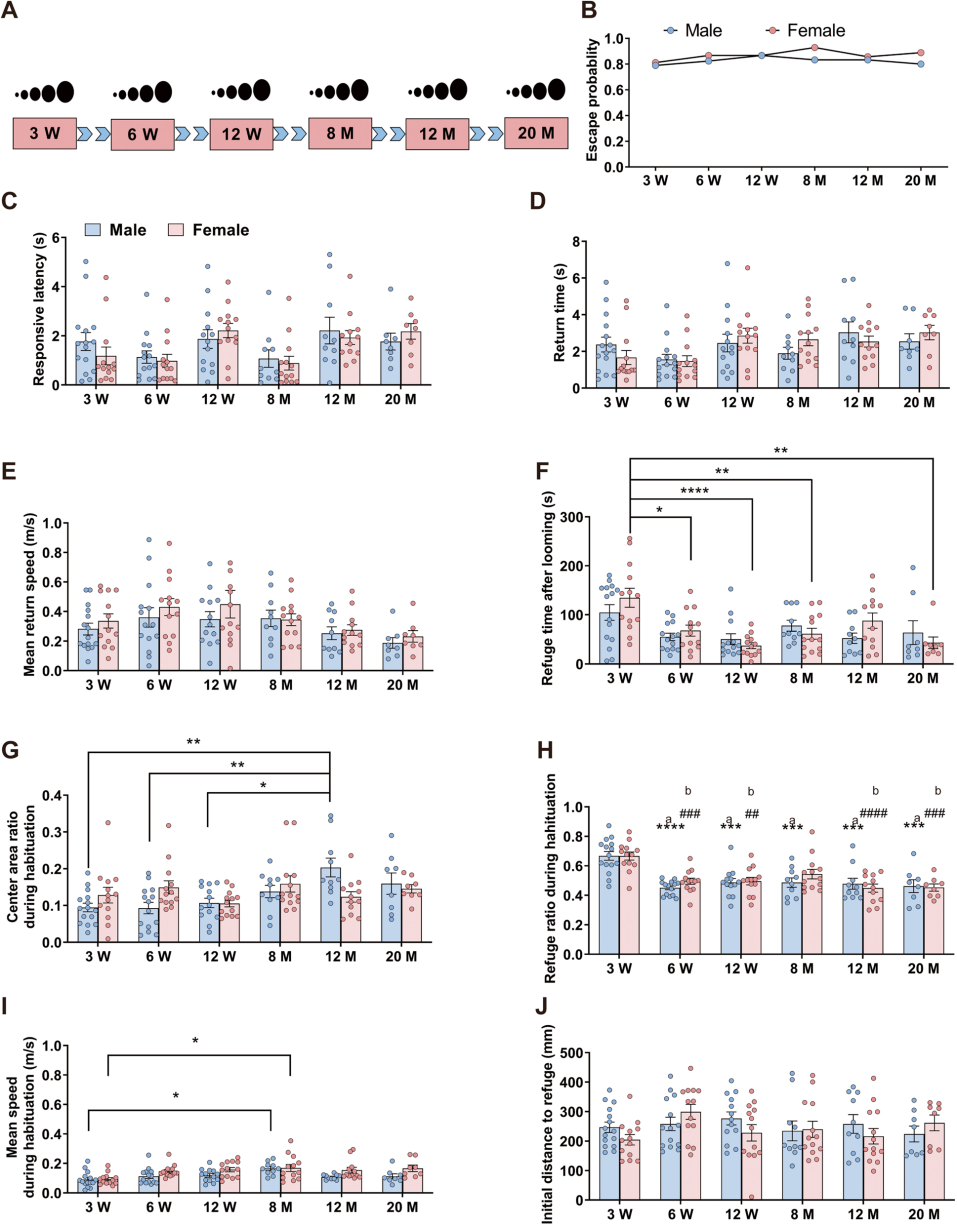
Both mouse sexes exhibit consistent visually-evoked innate fear behavior throughout life. **A** Experiment flow chart. **B** The escape probability of different mouse groups following looming stimuli. **C**–**J** Between-group comparisons of **C** the latency for mice to initiate flight behavior following looming stimuli onset, **D** the time taken to return to refuge following looming stimuli onset, **E** the mean return speed back to the refuge following looming stimuli onset, **F** the time spent in the refuge following looming stimuli onset, **G** the ratio of time spent in the center of the arena in the 5-min acclimation period, **H** the ratio of time spent in the refuge to time spent outside the refuge during the 5-min acclimation period, **I** the mean speed during the 5-min acclimation period, and **J** the initial location distance at stimuli onset to refuge following looming stimuli onset. (a) 3-week-old male mice vs. male mice of different age group (6 W, 12 W, 8 M, 12 M, and 20 M); (b) 3-week-old female mice vs. female mice of different age group (6 W, 12 W, 12 M, and 20 M). *p < 0.05; **p < 0.01; ***p < 0.001; ****p < 0.0001. ^##^*p* < 0.01; ^###^*p* < 0.001; ^####^*p* < 0.0001. Data are expressed as mean ± SEM

Certain cognitive dysfunctions [46, 47] and instinctive behaviors [21, 34, 48, 49] generally vary with age. We investigated whether the ability to successfully address life-threatening cues similarly varies over the lifespan in both sexes. We conducted looming tests on male and female C57BL/6 J mice in childhood, adolescence, early-to-middle adulthood, middle age, and old age (3 weeks, 6 weeks, 12 weeks, 8 months, 12 months, and 20 months; Fig. 5A). Looming stimuli triggered flight behavior in 80 ~ 90% of mice in each group (Fig. 5B). There were no differences between age groups or between sex in response latency (Fig. 5C), return time (Fig. 5D), mean return speed (Fig. 5E) and initial distance to refuge (Fig. 5J). We found, however, differences in behavior that were not directly related to defensive behavior. Three-week-old female mice spent more time in the refuge after looming than did other female age groups (*p < 0.05; **p < 0.01; **** *p* < 0.0001) (Fig. 5F). Moreover, in the 5-min acclimation period, 12-week-old males spent more time in the central area than 3-week-old males (**p < 0.01), 6-week-old males (**p < 0.01), and 12-month-old males (*p < 0.05) (Fig. 5G). Three-week-old mice of both sexes spent more time during 5-min acclimation period in the refuge time than other groups (Fig. 5H). Three-week-old females had lower mean speed during habituation than 8-month-old females (*p < 0.05), while 3-week-old males had lower mean speed during habituation than 8-month-old male mice (Fig. 5I). The fact that 3-week-old mice spent a longer time in the refuge during the 5-min acclimation period and more time following looming stimuli may not reflect enhanced fear behavior, but simply a more general preference for the safe refuge. In summary, these results suggest that visually-evoked innate fear behavior is highly conserved throughout the lifespan in both sexes.

## Discussion

Various innate behaviors, such as aggression in *Drosophila* [10, 50] and humans [51], mating in *Drosophila* [11] and mice [52], parenting behavior across the animal kingdom [25, 53], and social behavior [3], are dependent to some degree on sex to facilitate resource acquisition and enhance reproductive success, resulting in an advantage in species survival. In the present study, using a 3D-motion learning framework, we were surprised to find that, although post-threat coping strategies had subtle sex differences, mice had a consistent lifelong defensive behavior, independent of sex, in response to perceived life-threatening stimuli. Our findings suggest that these subtle sexually differentiated mechanisms do not modulate defensive ability when presented with life-threatening cues, indicating that such a vital and conserved defensive behavior is equally important for both sexes. However, these subtle sex differences found in post-threat coping strategies may help males and females respond appropriately to different external environments. Our finding provides a more comprehensive insight to further our understanding of the evolutionarily importance of innate fear behavior.

There is an increasing trend in the application of deep-learning-based analysis to quantify behavioral phenotypes as a way of upgrading and updating traditional approaches [38, 54–56]. Storchi et al. developed a method of obtaining 3D reconstructions of the mouse using five body landmarks and revealed that defensive behaviors are more stimulus specific than indicated by locomotion data [57]. This study, which was based on a hierarchical 3D-motion learning framework with sixteen body landmarks [38], included an in-depth between-sex comparison of 14 movements displayed following visually-evoked innate defensive behavior. We note, based on consideration of energy and reproduction requirements, that preyed-upon mice that successfully escaped the perceived predator not only escaped back to the refuge but also left the refuge after a period of time. (Fig. 4E). Since there is a lack of research on behavioral features after escape termination, we investigated the post-escape period, and suggest that it is part of the whole defensive behavior pattern. We found that female mice displayed fewer instances of freezing than male mice in the post-escape period, which echoes the finding that female rats exhibited more darting and less freezing following Pavlovian fear conditioning [2]. This finding indicates that, after reaching safety, female mice are quicker than males to begin exploring the external environment. We speculate that these subtle sex differences in post-threat coping strategies may help males and females to respond appropriately to different external environment.

Sexual dimorphism of behavior can be qualitative or quantitative in nature and arises from sexually differentiated neural circuits, which in turn are shaped by the varying hormonal, genetic, and epigenetic environments of males and females during development and adulthood [58]. For example, sex differences in parental behavior arise from sexual dimorphism of a cell-type-specific neural circuit [59], while gene expression and epigenetic regulation modulate innate and learned behavioral outputs [4]. Sex differences in affective disorders, such as major depression disorder, are largely characterized by differences in neuroplasticity, genetics, and neural networks [60]. The superior colliculus (SC) may be the origin of sexual dimorphism of innate defensive behaviors given that optogenetic stimulation of parvalbumin-positive neurons in the SC elicits longer escape times and shorter freezing times in female mice than in male mice [61]. However, there is no direct empirical evidence yet to support this. Our hierarchical 3D-motion learning framework provides a wealth of information required to reveal sex-divergent characteristics of innate fear behavior in addition to the acquisition of better basic data sets for further analysis which can facilitate understanding of behavioral variation in fear-related psychiatric diseases.

Innate defensive behaviors are vital for individual survival. Diverse sensory stimuli, such as olfactory threats from predator odor [62–65], the presence of predators [66] such as snakes [67], and visual threats such as looming stimuli [42], can all evoke innate defensive behaviors. The response to visual threats is important in the wild, as auditory or olfactory cues only reveal the presence of threats, whereas visual information can better convey the precise location of a predator and the likelihood of an attack [68]. Moreover, there is accumulating evidence showing that visually-evoked innate fear responses elicited during the protocol, which stem from sensory signals dynamics, are highly conserved across species, including humans [69], monkeys [70], cats [71], rodents [42], pigeons [72], amphibians [73], and zebrafish [74]. Our results indicate that the ability to maintain such a vital and conserved defensive behavioral output, whether it be evoked through vision or audition, is equally important for both sexes. In addition, this defensive ability to visual threats does not change throughout the mouse lifespan. However, sex differences are generally found in mental disorders associated with maladaptive fear behavior [75–77], suggesting that there may be sex-specific modulation in innate fear behavior. Future work will be directed to uncover the neural circuit mechanism underlying sexual dimorphism of innate defensive behavior. This may provide a viable approach to acquire information relevant to human disorders.

In rodents, sex differences in addition to estrous phase differences are most consistently found in learned fear behaviors during context re-exposure following cued or contextual fear conditioning [78–80]. Differences in emotion-related behavior and neuronal activity in brain areas such as the basolateral amygdala are found between different sexes and between estrous cycle phases [81]. Animals in different phases of the estrous cycle display different behaviors, such as anxiety-like behavior [82], cued fear extinction [81], motivated behavior [83], and predator-odor-induced fear behaviors [32]. However, our data demonstrate that, although mice in the diestrus phase spent more time in the central area of the open field than mice in the estrus phase in the 5-min acclimation period, there was no difference in visually-evoked innate fear behavior due to estrous cycle phase. These findings suggest that stable visually-induced innate defensive behaviors appear to be beneficial for female survival in different estrous phases, which may provide an advantage for mating and for the continuation of the species.

Cognitive dysfunction and alterations in emotional responses generally vary with age [46], including decreased locomotor activity, increased anxiety-like behavior [84], and decreased social behavior [34, 48]. Many instinctive behaviors that are guided by seeking advantages while avoiding injury display age dependence. For example, adult mice tend to be more aggressive than both pubescent and even younger mice [21], while aged mice display shortened nocturnal sleep duration, increased frequency of daytime naps, and decreased slow wave sleep compared to young and adult mice [35, 49]. In the current study, our findings reveal that there is no significant difference in visually-evoked innate fear across different ages between male and female mice. These results indicate that harboring the ability to survive life-threatening circumstances is evolutionarily dominant across the lifespan in both sexes, and hence the underlying neural circuitry and modulatory mechanisms underlying visually-triggered innate defensive behavior may be highly inherent and conserved during development.

To maximize individual survival probability, animals should optimally have innate behaviors to cope with environmental threats and to fulfill internal demands. Evidence has shown that rodents prioritize looming-stimuli-evoked defensive behavior over sleeping [68] and foraging [85]. Furthermore, mice can rapidly learn that repeated stimuli is non-threatening and then adapt, which induces cognitively controlled suppression of escape behavior following looming stimuli [86]. Here, our findings demonstrate sex- and age-independent outcomes of visually-evoked innate fear behavior. The evidence presented in this study, together with other work, leads us to speculate that the ability of animals to escape danger may predominate over other innate behaviors in challenging life-threatening situations. In addition, sex differences in post-threat coping strategies related to visually-triggered defensive behavior may provide insights into sex differences in stress susceptibility and resilience.

## Conclusions

The ability to successfully address potentially life-threatening cues, such as aerial predators, is highly conserved in mice and is independent of sex and age. However, post-threat coping strategies do depend on sex. This current work provides a comprehensive insight which furthers our understanding of the evolutionarily importance of innate fear behavior as well as sexually divergent fear-related psychiatric disorders.

## Methods

### Animals

Wild-type virgin female and male C57BL/6 J mice aged 8 weeks and 12 weeks were purchased used (Beijing Vital River Laboratory Animal Technology Co., Ltd. (Beijing, China; RRID: IMSR_JAX:000664). Younger mice (aged 3 or 6 weeks old during experiments) were bred and housed in a Specific Pathogen Free (SPF) environment laboratory. Mice to be used at 8, 12, and 20 months old were housed from 12 weeks old until they reached the appropriate age. Mice were housed at 22–25 °C in a relative humidity of 55%, on a circadian light–dark cycle of 12/12 h with *ad libitum* access to food and water. Experiments were conducted during the light phase between 8:00 am–14:00 pm.

To compare sexes in the visually-evoked innate fear defensive behavior test, a total of 99 mice aged 10–12 weeks were used. These included 15 males and 84 females, of which, 14 females were in proestrus, 41 in estrus, 10 in metestrus, and 19 in diestrus. A total of 36 mice aged 9–13 weeks (12 males, 13 sham females, and 11 castrated females) were used in the female castration experiment. For the 3D-motion framework experiment, a total of 60 mice (30 males and females) were used. Throughout all experiments, operators were blind to data analysis and unaware of group allocation. In the aging experiment, a total of 168 mice were used (19 males and 16 females in the 3-week-old group corresponded to weaning age; 17 males and 15 females in the 6-week-old group corresponded to adolescence; 15 males and 15 females in the 12-week-old group corresponded to early-adulthood; 12 males and 14 females in the 8 month-old group corresponded to middle-adulthood; 12 males and 14 females in the 12-month-old group corresponded to middle-age, and 10 males and 9 females in the 20-month-old group corresponded to old age).

### Looming behavioral test

We performed behavioral experiments to assess the ability of male and female at different ages to process life-threatening information using an innately aversive overhead expanding spot (looming task, as previously described [39]). On day one, each animal was habituated to the test arena for 10 min. This arena was an open-top acrylic cylinder (“open field,” 50-cm diameter), adjacent to an alley (“refuge,” 50 cm × 10 cm), with free access between compartments, and was enclosed by a 30-cm high wall (Fig. 1B). On day two, following 5 min of free exploration and habituation in the arena, animals were given one looming stimuli trial which was presented automatically when the mouse entered a circular trigger area (“central area,” 25-cm diameter), which was concentric with the open-field periphery [39]. In brief, the looming stimulus was a dark disk that expanded from 2° to 40° in 300 ms, maintained this size for 50 ms before disappearing, and was then repeated 15 times at 30-ms intervals [40]. After the looming behavioral test, vaginal smears were performed within 10 min to determine female estrous cycle, which was identified following microscopic Giemsa examination of vaginal smears.

### Auditory stimuli

The behavioral arena was the same arena used for the looming test. The arena was embedded in a soundproof box (inner box, 120-cm length × 80-cm width × 70-cm height; outside box 140 cm × 100 cm × 90 cm). Auditory stimuli (broadband white noise) had a noise intensity (1 ~ 64 kHz) at 80 dB SPL and lasted 5 s. This was generated using Matlab (RRID:SCR_001622) code and was delivered through an ultrasound speaker (Pettersson L400) which was calibrated (Sound level meter: Hangzhou Aihua AWA5661-W). On day one, each animal was habituated to the test arena for 10 min. On day two, following a 5-min acclimation period in the arena, animals were presented with the sound stimuli twice. Each was presented automatically when the mouse entered a circular trigger area (“central area,” 25-cm diameter), which was concentric with the open-field periphery.

### Identification of estrous cycle phase

In female rodents, the estrous cycle has 4 defined stages (proestrus, estrus, metestrus, and diestrus in a 4- to 5-day cycle) [87]. Vaginal cytology, using the vaginal smear method, can help to identify estrous cycle stage. Vaginal smears were conducted using small cotton-tipped swabs (Shanghai Bebixin Trading Co., Ltd.) wetted with 0.9% saline. After vaginal insertion of restrained female mice, the swab was gently rolled around and then removed before transferring the vaginal epithelial cells to a dry glass slide. Then, slides were stained with Giemsa Stain solution (Solarbio Life Sciences, G1010) according to manufacturer instructions. Different phases of the estrous cycle were identified using the following information: the proestrus phase contained epithelial cells that were predominantly nucleated with some cornified; the estrus phase contained epithelial cells that were mostly cornified; the metestrus phase contained a mixture of nucleated cells, cornified epithelial cells, and leukocytes observed together, and the diestrus phase contained polymorphonuclear leukocytes.

### Ovariectomy

Adult C57BL/6 J female mice (8 weeks, *n* = 26) were used to investigate looming-induced defensive behavior with or without ovaries. Ovariectomies or sham surgeries were performed on day one. Bilateral ovariectomies were carried out on female mice (castration group) under general anesthesia with sodium pentobarbital (80 mg/kg, i.p.). After shaving abdominal hair, mice were placed on their ventral surface. Under aseptic conditions, a single retroperitoneal incision in the dorsal skin and musculature was made laterally. The ovaries were found by gentle retraction of the uterine body and then they were excised. The incisions of muscle and skin were re-closed with absorbable sutures according to prior work [88]. For the sham group, mice underwent the same procedure without ovary excision. Vaginal smears were carried out to confirm success of ovariectomy for continuous 5 days from day 15 to day 19. Mice that were observed to have an estrous cycle were excluded from the group. Looming behavior tests were then performed at day 31 as described above (Fig. 1B).

### Behavioral analysis

The following behavioral indices were used: the trajectory of mice to the refuge, the center-periphery ratio during the 5-min acclimation period, the refuge-arena ratio in the 5-min acclimation period, the mean speed during the 5-min acclimation period, the initial distance to refuge and the mean return speed after looming stimuli. These were calculated using tracing data obtained from the infrared touchscreen data as reported previously [39]. We manually measured the responsive latency (latency to flight following stimuli), return time and time spent in the refuge after looming stimuli was triggered.

### Multi-view motion-capture device

The multi-view video capture device is shown in Fig. 2A and was described in detail previously [38, 39]. The arena used to monitor mouse behavior was a circular open field with a white plastic floor and transparent acrylic walls. The base diameter was 50 cm and the walls had a height of 30 cm. There was an adjacent alley (12 cm × 50 cm × 30 cm) and there was free access between compartments. The circular open field was placed at the center of a 130 × 130 × 90 cm movable, stainless-steel support framework. Four Intel RealSense D435 cameras were mounted orthogonally on the four supporting pillars of the shelf. Mice were allowed to freely explore the circular open field for 5 min, after which looming stimuli was triggered manually when the mouse entered a circular trigger area (diameter 25 cm). Each mouse was tested in only one experimental condition and had 3–5 effective trials in order to acquire more information in one session. However, we only chose one trial, the first trial in most cases. The detailed methods of this 3D multi-view motion-capture system setup (BA-DC01, Shenzhen Bayone BioTech Co., LTD, Shenzhen) and analysis was described in our previous work [38] (Additional file 7: Figure S3).

### 3D-motion learning framework

The method (3D-motion learning framework) and the self-developed software (Behavior Atlas) we used to quantify defensive behavioral phenotypes adopted a parallel motion decomposition strategy. Complex mammalian behavior includes locomotion and non-locomotor movement (NM) [89]. Locomotion can be represented by velocity-based parameters. NM is manifested by movement of the limbs or organs without movement of the torso and is controlled by many degrees of freedom. Therefore, we used the dynamic time alignment kernel (DTAK) to measure the similarity between NM segments and then separated data by NM features. High-dimensional NM features were reduced to 2D NM space by uniform manifold approximation and projection (UMAP), which correspond to UMAP1 and UMAP2. Finally, together with the additional velocity-based locomotion dimension, unsupervised clustering was applied to the 3D behavioral features space and the classification of behavioral types. Based on the above methodological background, and due to the inconsistent metric scales between velocity and UMAP components, we normalized the velocity dimension using the Z-score method for the purpose of better visualization. Negative normalized velocity was not used in the current study. The input dimensions of the two feature space panels were the same. The feature space in Fig. 2B shows 40 good-quality unsupervised clustering behavioral phenotypes by machine learning. And the feature space in Fig. 2C shows good quality clustering of 12 supervised learning-labeled behavioral movements. The feature space shows that both the 40 behavioral phenotypes and 12 behavioral movements were well separated. To visualize the high-dimension non-locomotion features, UMAP was used for dimensionality reduction. We combined UMAP1, UMAP2, and locomotion features for unsupervised clustering.

### Statistical analysis

All data are presented as means ± the standard error of the mean (SEM) and were analyzed using GraphPad Prism 8 (RRID:SCR_002798). One-way analysis of variance (ANOVA) was used in the experiment comparing males and females including estrous phase analyses, castration experiments, and auditory experiments. Two-way ANOVA was used for the 3D-motion capture experiment. Holm-Sidak’s multiple comparisons test was applied as a post hoc analysis if needed and p values were deemed statistically significant if *p* < 0.05. Two-way ANOVA was used for between-subject factors of age (3 weeks old, 6 weeks old, 12 weeks old, 8 months old, 12 months old, and 20 months old) and sex. Tukey’s multiple comparisons test was applied as a post hoc analysis if needed and *p* values were deemed statistically significant if *p* < 0.05.

## Supplementary Information

**Additional file 1: Movie S1.** A representative video showing the looming behavioral test.

**Additional file 2: Figure S1.** Elimination of estrous cycle in female mice did not affect defensive behavior following exposure to looming stimuli.

**Additional file 3: Figure S2.** There were no sex and estrus-phase differences in auditory-induced defensive behavior.

**Additional file 4: Movie S2.** A video showing the auditory stimuli behavioral test.

**Additional file 5: Movie S3.** A video showing a male mouse after looming stimuli from the 3D motion capture setup.

**Additional file 6: Movie S4.** A video showing a female mouse after looming stimuli from 3D motion capture setup.

**Additional file 7: Figure S3.** Workflow of data acquisition and analysis of 3D motion multi-layered framework.

**Additional file 8: Table 1.** Definition of movements and statistical test results.

**Additional file 9: Figure S4.** There were no sex differences in flight responses or in behavior state transition following looming stimuli.

## Data Availability

All the raw videos associated with the looming behavior test data for 3D-motion learning framework analysis in Figs. 2, 3, and 4 and Additional file 4–9 are available in the Zenodo repository [90] https://doi.org/10.5281/zenodo.7243193. Any other relevant data that support the findings of this study are available from the corresponding author upon reasonable request. The 3D multi-view motion-capture system setup and its related software are, however, available from the authors upon reasonable request and with permission of Shenzhen Bayone BioTech Co., LTD (http://behavioratlas.cn/). The methods and code described in this paper (see below), as well as BehaviorAtlas original paper (Huang et al. Nat Commun, 2021) are freely available for not-for-profit academic researchers.

## Code availability

The original codes of 3D framework can be available on Zenodo [91] https://doi.org/10.5281/zenodo.4626951. Post analysis code for the looming behavior test data for 3D-motion learning framework are available on Zenodo [92] https://doi.org/10.5281/zenodo.7243737.
