# Supplementary material for: Male and female mice display consistent lifelong ability to address potential life-threatening cues using different post-threat coping strategies
Source: BMC Biol. 2022 Dec 15;20:281. doi: 10.1186/s12915-022-01486-x (PMC9753375; doi:10.1186/s12915-022-01486-x)
Supplement: Supplementary file 2 — Additional file 2: Figure S1. Elimination of estrous cycle in female mice did not affect defensive behavior following exposure to looming stimuli. [file 12915_2022_1486_MOESM2_ESM.docx]

**Additional file 2: Figure S1. Elimination of estrous cycle in female mice did not affect defensive behavior following exposure to looming stimuli.**


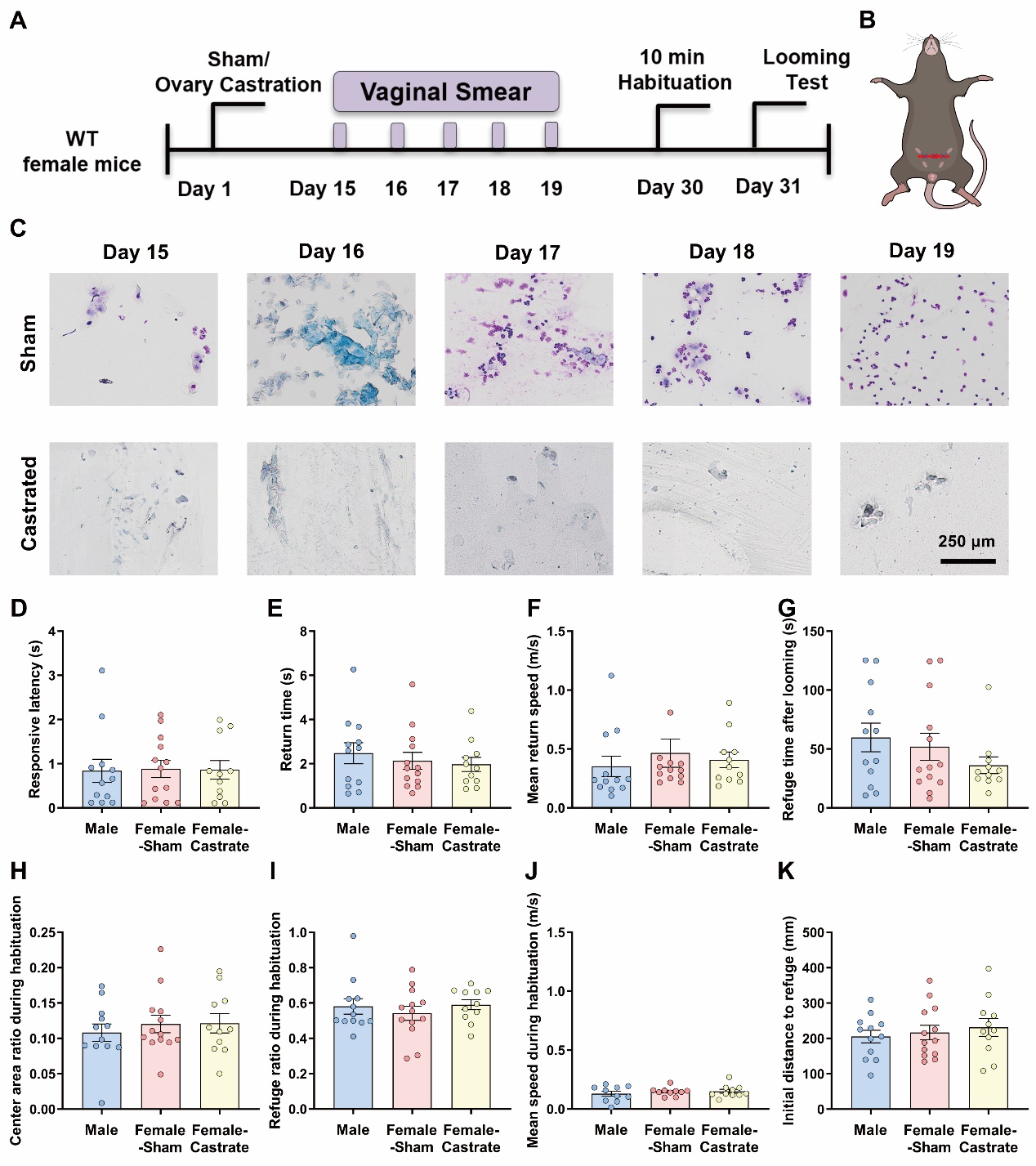


**A**, Experimental flow chart. **B**, Schematic diagram of the ovariectomy. **C**, Vaginal cytology identifying estrous cycle elimination in the castrated group compared to the sham group. **D**, Latency of mice to initiate flight behavior following looming stimuli onset. **E**, Latency of mice to return to the refuge following looming stimuli onset. **F**, Mean speed of return to the refuge following looming stimuli onset. **G**, Time spent in the refuge following looming stimuli onset. **H**, The ratio of time spent in the center area during the 5 min acclimation period. **I**, The ratio of time spent in the refuge during the 5-min acclimation period. **J**, Mean speed during the 5-min acclimation period to the arena. **K**, The initial location distance at stimuli onset to refuge following looming stimuli onset. Scale bar, 250 μm. Data are expressed as mean ± SEM.
