# Supplementary material for: Male and female mice display consistent lifelong ability to address potential life-threatening cues using different post-threat coping strategies
Source: BMC Biol. 2022 Dec 15;20:281. doi: 10.1186/s12915-022-01486-x (PMC9753375; doi:10.1186/s12915-022-01486-x)
Supplement: Supplementary file 3 — Additional file 3: Figure S2. There were no sex and estrus-phase differences in auditory-induced defensive behavior. [file 12915_2022_1486_MOESM3_ESM.docx]

**Additional file 3: Figure S2. There were no sex and estrus-phase differences in the auditory-induced defensive behavior.**


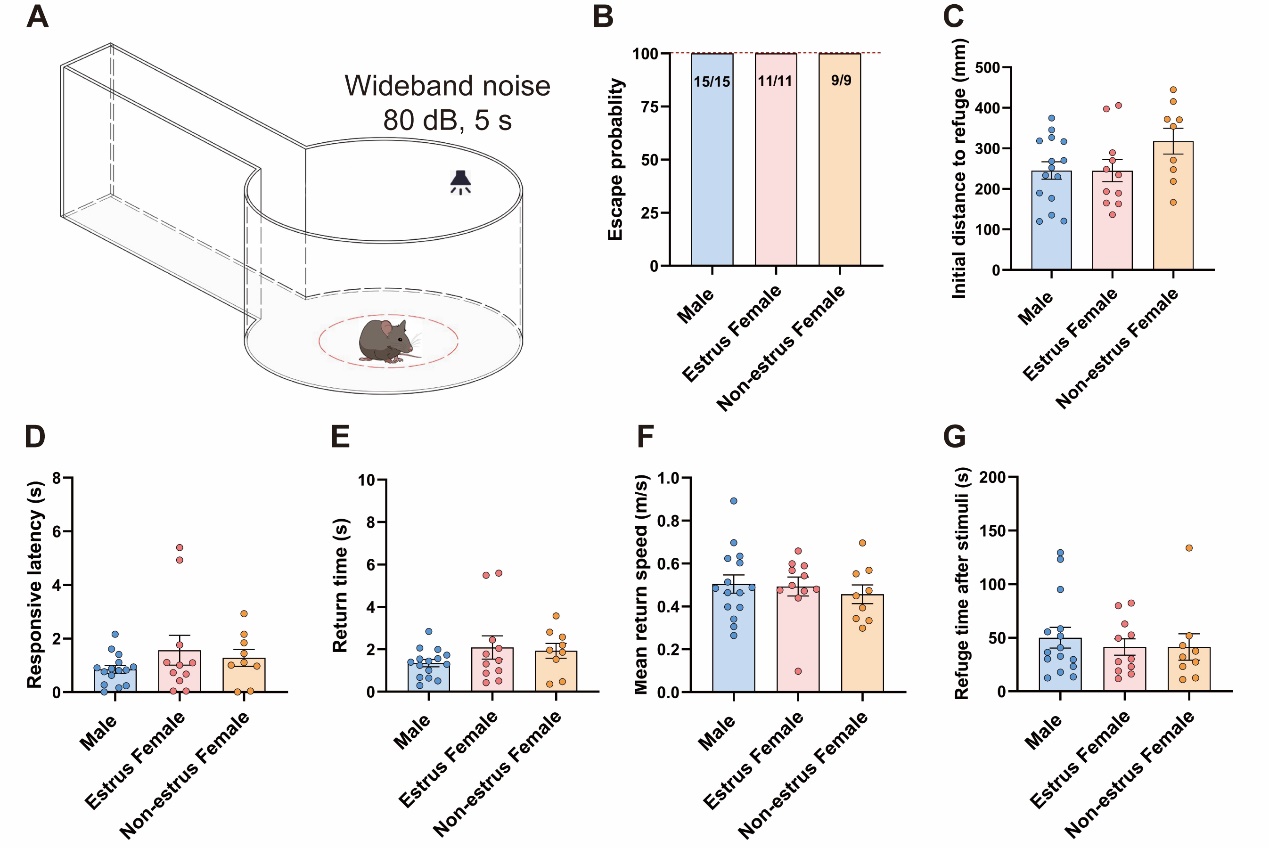


**A**, Schematic diagram of auditory-induced defensive behavior: a cylindrical open field, a rectangular refuge, and an infrared touchscreen frame below. Auditory stimuli were presented automatically in the floor of the setup. **B**, Escape probability after auditory stimuli. **C**, The initial location distance at stimuli onset to refuge following auditory stimuli onset. **D**, Latency of mice to initiate flight behavior following auditory stimuli onset. **E**, Latency of mice to return to the refuge following auditory stimuli onset. **F**, Mean speed of return to the refuge following auditory stimuli onset. **G**, Time spent in the refuge following auditory stimuli onset. Data are expressed as mean ± SEM.
