# Supplementary material for: Male and female mice display consistent lifelong ability to address potential life-threatening cues using different post-threat coping strategies
Source: BMC Biol. 2022 Dec 15;20:281. doi: 10.1186/s12915-022-01486-x (PMC9753375; doi:10.1186/s12915-022-01486-x)
Supplement: Supplementary file 7 — Additional file 7: Figure S3. Workflow of data acquisition and analysis of 3D motion multi-layered framework. [file 12915_2022_1486_MOESM7_ESM.docx]

**Additional file 7: Figure S3. Workflow of data acquisition and analysis of 3D motion** **multi-layered framework.**


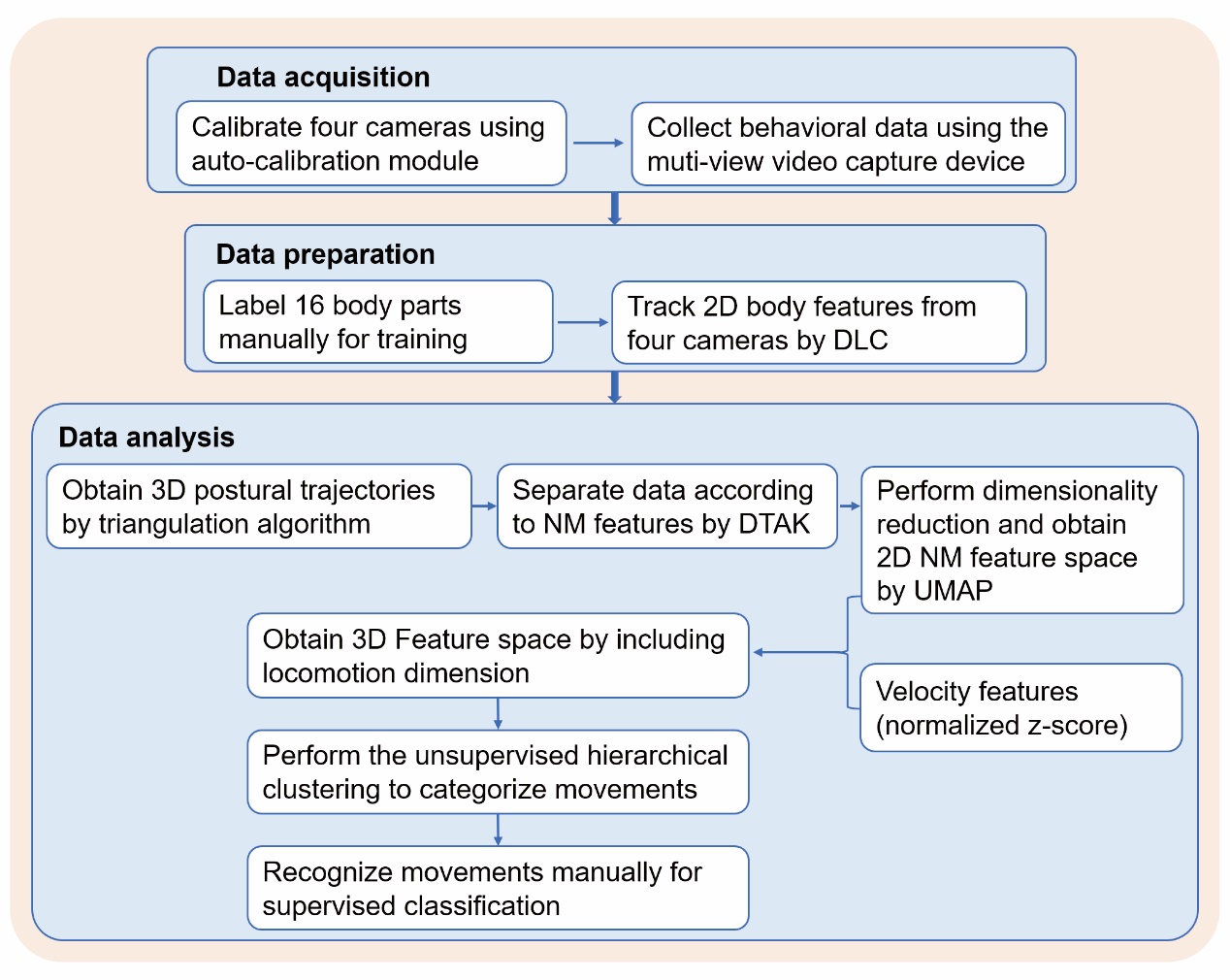


2D, two-dimensional; 3D, three-dimensional; DLC, DeepLabCut; DTAK, dynamic time alignment kernel; NM, non-locomotor movement; UMAP, uniform manifold approximation and projection.
