# Supplementary material for: Male and female mice display consistent lifelong ability to address potential life-threatening cues using different post-threat coping strategies
Source: BMC Biol. 2022 Dec 15;20:281. doi: 10.1186/s12915-022-01486-x (PMC9753375; doi:10.1186/s12915-022-01486-x)
Supplement: Supplementary file 9 — Additional file 9: Figure S4. There were no sex differences in flight responses or in behavior state transition following looming stimuli. [file 12915_2022_1486_MOESM9_ESM.docx]

**Additional file 9: Figure S4. There were no sex differences in flight response and behavior state transition following looming stimuli.**


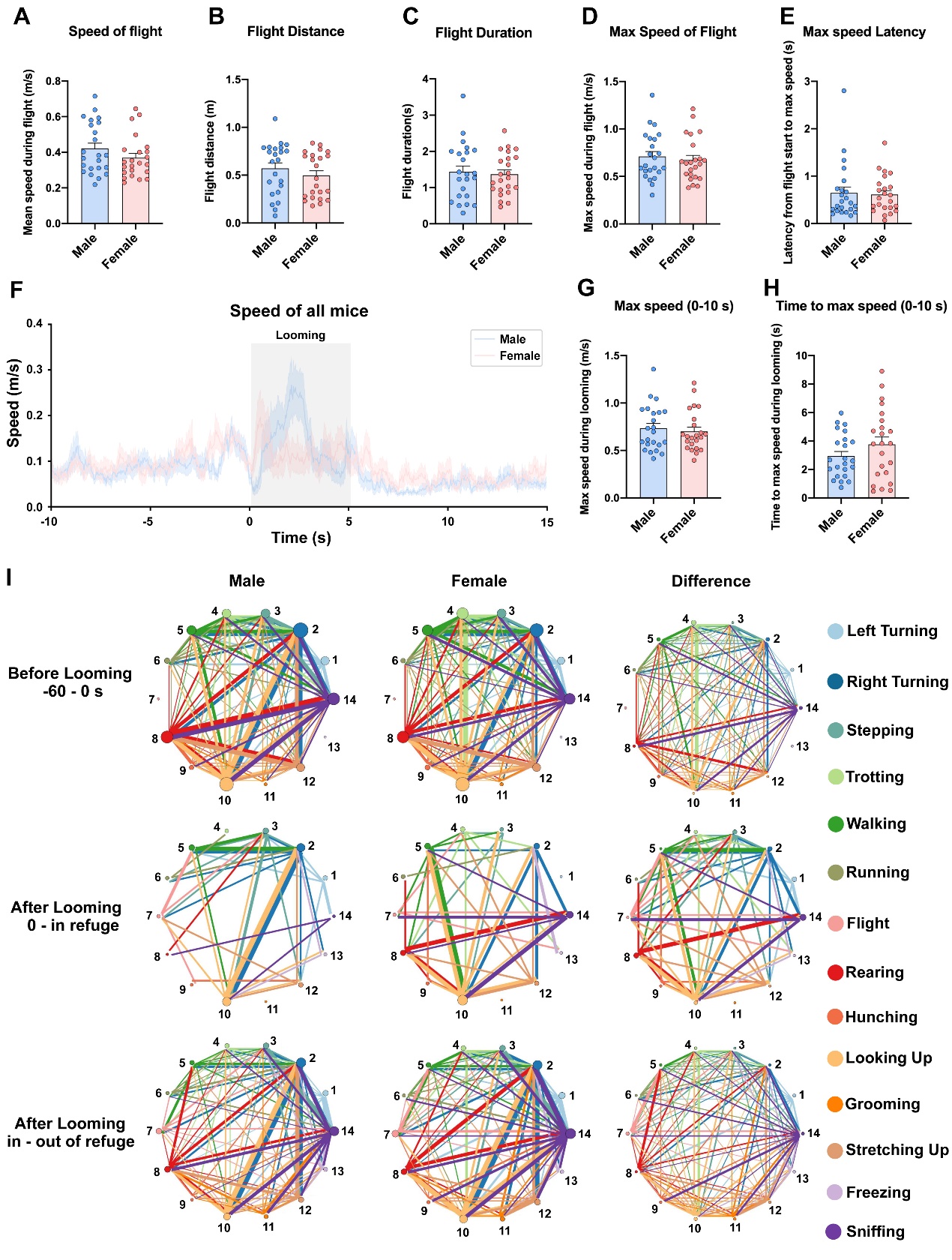


**A**, Mean speed during flight. B, Total flight distance. C, Total duration of flight. D, Maximum speed during flight. E, Duration from onset of flight to maximum speed. F, Real-time flight speed. G, Maximum speed during looming stimuli. H, Latency to maximum speed during looming. Data are expressed as mean ± SEM. I, State transitions of the movement modules from -60 s before looming to timepoint at which mouse went out of refuge and differences in the state transitions between male and female mice. Data are expressed as mean ± SEM.
